# Changes to intraocular pressure and its correlation with corneal diameter in infants aged from 0 to 36 months

**DOI:** 10.3389/fped.2022.954337

**Published:** 2022-10-10

**Authors:** Jian-Cang Wang, Fei-Fan Du, Shuo-Shuo Meng, Yun-Shuo Wei, Xi-Ting Guo

**Affiliations:** Department of Ophthalmology, Hebei Children’s Hospital, Hebei Medical University, Shijiazhuang, China

**Keywords:** infants, intraocular pressure, corneal diameter, correlation, ophthalmology

## Abstract

**Objective:**

This study examines the distribution and development of intraocular pressure (IOP) in infants aged from 0 to 36 months and analyzes its correlation with corneal diameter.

**Methods:**

The study used a retrospective case analysis methodology. Healthy infants treated in the ophthalmology department of Hebei Children's Hospital from December 2012 to December 2020 were included in the study. Among these infants, 385 had their IOP measured, and 432 had their corneal diameters measured. Furthermore, information such as birth history, growth and development, IOP, and corneal diameter were collected. Their IOPs were measured with an iCare portable rebound tonometer when the child was awake and calm, and the corneal diameter was measured with a Castroviejo caliper under chloral hydrate sedation. The infants were divided into five groups according to age, and SPSS statistical software was used to analyze, compare, and correlate IOP and corneal diameter variations.

**Results:**

The mean IOP values of 0–1 month, 1–6 months, 6–12 months, 12–24 months and 24–36 months groups were 7.42 ± 1.92, 9.10 ± 2.85, 12.00 ± 3.15, 13.72 ± 3.09, and 15.14 ± 2.67 mmHg, respectively. The differences in IOP of the 0–1 month old infants and the 1–6 months old infants with the other three groups were statistically significant; the difference in IOP between the 6–12 months group and the 24–36 months group was statistically significant. In the studied groups, the horizontal corneal diameters were 9.78 ± 0.14, 10.50 ± 0.29, 10.86 ± 0.23, 11.38 ± 0.07, and 11.72 ± 0.04 mm, respectively, and the vertical diameters of the cornea were 9.28 ± 0.26, 10.07 ± 0.18, 10.28 ± 0.14, 10.56 ± 0.24, and 10.85 ± 0.03 mm, respectively. The differences in the vertical and horizontal diameters of the cornea among the groups were statistically significant.

**Conclusion:**

Infants' IOP and corneal diameter positively correlate with age, and they peak in the first 12 months.

## Introduction

The diagnosis of glaucoma in children poses intricate problems. The intraocular pressure (IOP) and the size of the cornea is important in the diagnosis of primary infantile glaucoma. Infants' eyes are continually growing and developing, and their corneal thickness and scleral hardness are significantly different from those of adults (1). Therefore, the IOP range of adults cannot be used to evaluate whether an infant's IOP is normal (2). Because infants and young children cannot cooperate with relevant examinations and the examination status is different, the measured IOP values change greatly (3). Establishing a baseline IOP value for infants and young children and understanding how it is different could provide a reference for measuring their eye growth and development; it could also enable the early diagnosis of glaucoma.

## Subjects and methods

### Subjects

Study subjects: Infants aged from 0 to 36 months with complete medical records who underwent fundus screening in the ophthalmology department of our hospital from December 2012 to December 2020. All data were obtained from the measurement of the right eye.

Included subjects: 1. Infants carried to full-term without a history of eye disease and no record of hereditary eye disease in the family. 2. Healthy infants with no eye lesions confirmed by examination.

Excluded subjects: 1. Infants born prematurely or those with congenital ocular dysplasia. 2. Infants with incomplete hospital records. 3. Infants with eye diseases that affect IOP, such as retinal detachment or cataracts, and those that had previous eye surgeries. 4. Other infants whose eye data could not be collected.

## Methods

Measuring IOP: The IOP is diurnal and rhythmic, which varies at different times of the day. It is higher in the early morning and lower in the evening. All examinations were completed between 10 a.m. and 1 p.m. All cooperative participants sit quietly on their parent's lap. The measurement was performed when the child was calm, not crying, eyes open, and without assistance. If the subject was awake and did not need anesthesia to stay calm, the examination was carried out by an experienced nurse. If necessary, the nurse gently separated the eyelids. The IOP was measured with an iCare (IC200) rebound tonometer with a new sterile probe for each subject. The forehead support was disinfected with alcohol cotton balls before and after each operation. The length of the support rod was adjusted until the pressure measuring head was 4–8 mm away from the apex of the cornea. With the probe facing the center of the cornea, the tonometer was activated. After recording a measurement, the tonometer automatically calculated its *P*-value. This was repeated three times, and the mean of the resultant measurements was considered the subject's IOP.

Measuring corneal diameter: The subjects were given chloral hydrate as a sedative and anesthetic Proparacaine eye drops after the IOP measurement. Then, an eye speculum was used to fully expose the cornea. The horizontal and vertical diameters of the cornea were measured three times with a Castroviejo caliper, and the mean was considered the dimensions.

Statistical analysis: Data were analyzed using SPSS 20.0 statistical software. A variance homogeneity test was conducted on the IOP and corneal diameter for all groups. A rank sum test was used for a pairwise comparison among the groups if the variance was heterogeneous, and a *P*-value of less than 0.05 was considered statistically significant.

## Results

### Overview

Healthy infants who underwent fundus screening in the ophthalmology department of our hospital from December 2012 to December 2020 were included in this study; subjects were grouped according to their age in months. Of the subjects, 385 had their IOP measured. There were 71, 83, 79, 75, and 77 subjects in the 0–1 month, 1–6 months, 6–12 months, 12–24 months, and 24–36 months groups, respectively; 190 of them were girls, and gender did not significantly affect the results. Four hundred thirty-two subjects had their corneal diameters measured. There were 82, 102, 92, 80, and 72 in the 0–1 month, 1–6 months, 6–12 months, 12–24 months and 24–36 months groups, respectively; 223 of them were girls, gender did not significantly affect the results.

## Intraocular pressure and correlation analysis

Mean and standard deviations were used to express IOP, and the data are summarized in Table 1. The subjects' IOP increases gradually with increasing age. The data were tested for variance homogeneity and found to have variance heterogeneity with a chi-square of 31.1918. A rank sum test was performed on the data among the groups and showed no significant differences in IOP between the following groups (*P* > 0.05): 0–1 month and 1–6 months; 6–12 months and 12–24 months; and 12–24 months and 24–36 months. However, there were significant differences in IOP between the following groups (*P*< 0.05): 0–1 month and the other three groups; 1–6 months and the other three groups; and 6–12 months and 24–36 months.

**Table 1:** Intraocular pressure measurement results (*χ* ± S, mmHg).

Group	0–1	1–6	6–12	12–24	24–36
Intraocular pressure	7.42 ± 1.92	7.76 ± 2.02	12.00 ± 3.15	13.72 ± 3.09	15.14 ± 2.67

## Vertical and horizontal corneal diameter and correlation analysis

Mean and standard deviations were used to describe the vertical and horizontal corneal diameters, and the data are summarized in Table 2. The subjects’ vertical and horizontal corneal diameters increase gradually with increasing age. The data were tested and were found to have variance heterogeneity. A rank sum test was performed on the data and showed significant differences in the vertical and horizontal corneal diameters (*P* < 0.05).

**Table 2:** Corneal diameter measurement results (*χ* ± S, mmHg).

Group	0–1	1–6	6–12	12–24	24–36
Horizontal corneal diameter	9.78 ± 0.14	10.50 ± 0.29	10.86 ± 0.23	11.38 ± 0.07	11.72 ± 0.04
Vertical corneal diameter	9.28 ± 0.26	10.08 ± 0.18	10.28 ± 0.14	10.56 ± 0.24	10.85 ± 0.03

## Discussion

The IOP measurement is an important index for diagnosing and treating ocular hypertension, ametropia, infant glaucoma, and other diseases. Goldmann applanation tonometry has been the golden standard for measuring intraocular pressure for decades. However, infants are not cooperative and IOP measurement with the Goldmann tonometer is difficult. Although there are several other instruments, such as Perkins hand-held applanation tonometer and pneumatonometers, they often require general anesthesia in infants and children. However, due to the nature of infants and young children, they are unlikely to cooperate during examinations, making measuring their IOP difficult; this leads to large differences in IOP measurements between those that are anesthetized and those that are not (4). A number of studies have shown that the measurement results of Goldmann tonometer and Icare tonometer have good consistency, and Icare tonometer is more convenient for measuring the intraocular pressure of infants and young children, so Icare tonometer was selected for measurement (5–7).

There are few foreign or domestic studies on measuring the IOP in infants and young children. Understanding the normal IOP level of infants and young children at different ages and how it changes with age is important for the early detection of glaucoma in infants and young children, postoperative IOP control, and preventing complications. As there is no reliable benchmark for IOP in infants and young children in China, this study was undertaken.

In this study, IOP was measured between 10 a.m. and 1 p.m., and the influence of measurement time on IOP was excluded. In addition, measuring IOP in infants can be affected by factors such as crying or non-cooperation, which could increase IOP. Furthermore, the IOP value is lower than normal under general anesthesia or sedation (8). Therefore, the IOP was measured without anesthesia and relied on the subjects' cooperation in this study; the resultant data were baseline IOP values taken in an ideal state.

The study showed that healthy infants have lower IOP than adults and that IOP positively correlates with age. It also showed a significant difference in IOP between the 0–1 month group and all the other groups except the 1–6 months group. Therefore, it is speculated that the IOP of children grows rapidly from birth to six months and increases significantly after that. The difference in IOP between the 6–12 months and 24–36 months groups was significant, but there was no significant difference between the 12–24 months and 24–36 months groups. Although infant IOP increases from 12 months, it is speculated that it does so more slowly than before. Previous studies have resulted in inconsistent infant IOP measurements. Possible reasons include subject selection criteria (such as premature delivery or not), type of tonometer, cooperation level of the infants and young children, and the anesthetic method. Moussa et al. (9) observed that the mean IOP at birth was 9.59 ± 2.3 mmHg. This study showed that the mean IOP of infants aged 0–1 month was 7.42 ± 1.92 mmHg, slightly lower than at birth, possibly because the infants were calmer. Radtke and Cohan (10) measured the IOP of 60 newborns from 19 to 173 h after birth, resulting in an average IOP of 11.4 ± 2.4 mmHg. Further analysis showed that increasing age did not affect IOP change. However, another study showed that the mean IOP of premature infants with low birth weight was 14.9 ± 4.5 mmHg, and the IOP decreased by 0.29 mmHg every additional gestational week (11). This result contradicted the above results; it is still uncertain whether it is related to premature delivery. Similar to this study, Gharebaghi et al. (12) reported that the IOP of children under 12 years increased with age. This study showed that the IOP of infants was positively correlated with age; within 36 months old, IOP increased with increased age, and the peak IOP increase was from birth to 12 months. The IOP increased rapidly with increased age, and it had nearly reached the adult level at 12 months old. However, infant IOP increased slowly with age after 12 months.

In this study, the horizontal corneal diameter of newborns was 9.78 ± 0.14mm, and the vertical diameter was 9.28 ± 0.26mm. The horizontal and vertical corneal diameters positively correlated with age. Studies on corneal development are also inconsistent when reporting how long is needed for corneas to mature (13). Ozdemir et al. (14) believed that the horizontal corneal diameter of normal newborns was 10–10.5mm, which increased by 0.15–1.0mm at the end of one year; this study's results support this. Nanda (15) believed that the corneal diameter of infants 6–12 months old could approach the normal adult diameter of 11.80mm. Other studies demonstrated that (16) the corneal diameter of two-year-old infants was close to that of adults. Some studies (17) suggested that infant corneal development continues after the first five years. In this study, a Castroviejo caliper was used to measure the horizontal and vertical corneal diameters, and the development of corneal diameter positively correlated with both IOP and age. The increases were fastest from birth to one year old, and it is speculated that this is the critical period for infant eye development. The corneal diameter and IOP increase slowly from one to three years old, approaching an adult level.

There are reported differences in the time taken for IOP and corneal diameter to mature; however, like other body structures, it is believed that neonatal IOP and corneal diameter grow and develop until they mature. In this process, the development of IOP and corneal diameter should correlate with the growth of the ocular axis (18, 19). Children are born with a short ocular axis and have hyperopia. The ocular axis develops with age, with the fastest growth of approximately 0.5–0.6mm per year in the first two to three years. After that, growth gradually slows and stabilizes between six and seven years old (20). Infants' eyes are elastic because the cornea and sclera are composed of elastic collagen fibers. As the IOP increases, it acts biomechanically on immature connective tissues (collagen fibers), expanding the cornea and lengthening the ocular axis (21, 22). Therefore, it is speculated that an increased IOP may be the driver of corneal enlargement and ocular axis lengthening.

Glaucoma in children is characterized by an elevated IOP and optic disk depression. In addition, glaucoma in infants and young children is often accompanied by an increase in corneal diameter. Therefore, IOP, corneal diameter, the cup/disk ratio (C/D) value, and axial length may be clinically important for diagnosing glaucoma in infants and young children. If two of the three exceed normal ranges, the likelihood of infants and young children suffering glaucoma is greatly increased. There are three possible situations: (1) An increased IOP and corneal diameter indicate that a continuously high IOP increases corneal diameter. Due to the elasticity of the eyes of infants and young children, a high IOP may not have damaged the optic disk, but glaucoma should be suspected. (2) An elevated IOP and increased C/D value are characteristics of glaucoma. (3) An increased corneal diameter and C/D value may be due to the elasticity of the eyeball, the compensatory lengthening of the ocular axis, and the elevated IOP is not shown. Therefore, it is crucial to determine IOP and corneal diameter growth and development for diagnosing, treating, and preventing congenital glaucoma.

However, this study had some limitations. For example, due to the problems involved with getting infants to cooperate, such factors including corneal thickness, ocular axis, and refractive status were note measured. These factors could affect IOP and should be studied in future research.

## Conclusion

This study reported IOP values at different ages, from birth to three years old; this is rare in foreign and domestic studies. Moreover, the subjects were not anesthetized during the examination; this ensured that the measured IOP was a reliable indicator of the children's physiological state. The iCare rebound tonometer is easy to use, does not require topical anesthesia, and is well tolerated by cooperative infants. It was used in upright and lateral positions and was well tolerated. The iCare rebound tonometer and Goldmann applanation tonometer measure IOP consistently. Therefore, this study's findings reflect that the iCare rebound tonometer is a reliable instrument for measuring IOP in infants.

## Data Availability

The original contributions presented in the study are included in the article/Supplementary Material, further inquiries can be directed to the corresponding author/s.
